# Supplementary material for: Temporal dynamics and microbial interactions shaping the gut resistome in early infancy
Source: Nat Commun. 2025 Aug 30;16:8139. doi: 10.1038/s41467-025-63401-6 (PMC12398494; doi:10.1038/s41467-025-63401-6)
Supplement: Supplementary file 1 — Supplementary Information [file 41467_2025_63401_MOESM1_ESM.pdf]

SUPPLEMENTARY INFORMATION

Temporal dynamics and microbial interactions shaping the gut resistome in early infancy

Ioanna Chatzigiannidou[1], Pi L. Johansen[1], Rasmus K. Dehli[1], Janne Marie Moll[1], Carsten Eriksen[1], Pernille N. Myers[1], Henrik M. Roager[2], Lili Yang[3], Jakob Stokholm[4,5], Søren J. Sørensen[3], Karen A. Krogfelt[6,7], Martin F. Laursen[8], Urvish Trivedi[3,4], Annika Scheynius[9,10], Karsten Kristiansen[3], Axel Mie[9,11], Johan Alm[9,10], Susanne Brix[1*]

1. Department of Biotechnology and Biomedicine, Technical University of Denmark, 2800 Kgs. Lyngby, Denmark
2. Department of Nutrition, Exercise and Sports, University of Copenhagen, 1958 Frederiksberg C, Denmark
3. Department of Biology, University of Copenhagen, 2100 Copenhagen, Denmark
4. Copenhagen Prospective Studies on Asthma in Childhood, Herlev and Gentofte Hospital, University of Copenhagen, 2820 Gentofte, Denmark
5. Department of Food Science, University of Copenhagen, 1958 Frederiksberg C, Denmark
6. Department of Bacteria, Parasites and Fungi, Statens Serum Institut, 2300S Copenhagen, Denmark
7. Department of Science and Environment, Roskilde University, 4000 Roskilde, Denmark
8. National Food Institute, Technical University of Denmark, 2800 Kgs. Lyngby, Denmark
9. Department of Clinical Science and Education, Karolinska Institutet, Södersjukhuset, SE-118 83 Stockholm, Sweden
10. Sachs' Children and Youth Hospital, Södersjukhuset, SE-118 83 Stockholm, Sweden
11. Department of Environmental Science, Stockholm University, SE-106 91 Stockholm

* Corresponding author: sbrix@dtu.dk

Keywords: resistome, early life, gut microbiome, aromatic lactic acids, antimicrobial resistance

This file contains:

Supplementary Figures 1-9

Supplementary Tables 1-9

Supplementary Data 1-4 are supplied as separate spreadsheet files

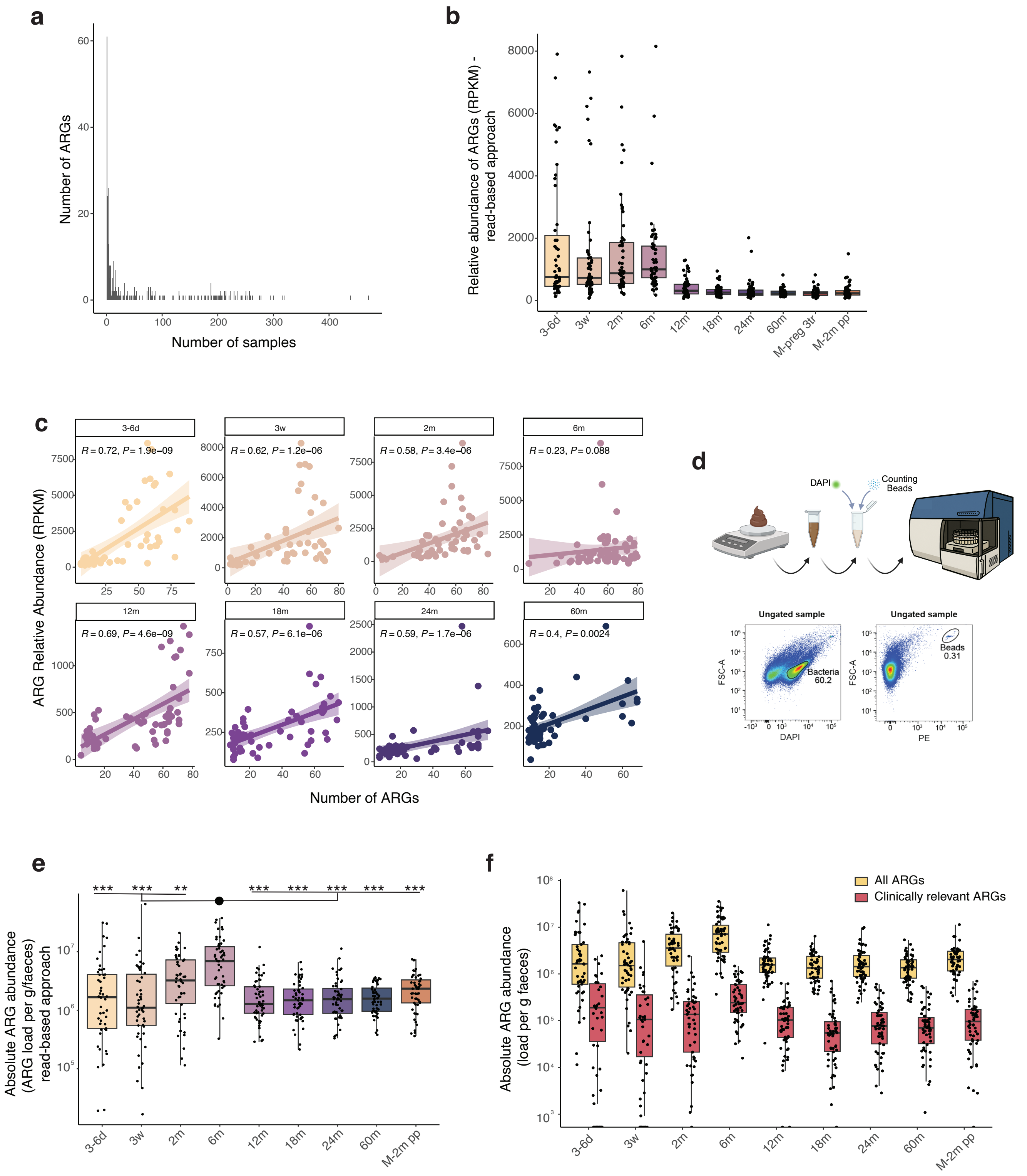

Supplementary Fig. 1| ARG load dynamics. a, Histogram depicting the number of samples with a specific ARG richness. **b,** Relative abundance of ARGs (RPKM) as defined by the read-based method across time points. **c,** correlation of the ARG richness with the ARG relative abundance. **d,** Flow cytometry was used to determine the DAPI+ bacterial population to calculate the bacterial load per gram of faeces. The fecal sample preparation protocol was designed using BioRender (https://BioRender.com/v8j8si4). The photo of a BD FACSCanto^{TM} Flow Cytometer was converted into a drawing using ChatGPT (OpenAI), while the two scatter plots depict actual data generated in this study. **e,** absolute ARG abundance (RPKM/10^6 x bacteria per gram of faeces) as defined by the read-based method across time points. **f,** Absolute ARG abundance (contig-based) of the total ARGs and the clinically relevant ARGs. **a,b,e,f**: 3-6d (*n* = 51), 3w (*n* = 52), 2m (*n* = 55), 6m (*n* = 55), 12m (*n* = 56), 18m (*n* = 55), 24m (*n* = 56), 60m (*n* = 55), M-preg 3tr (*n* = 56), M-2m pp (*n* = 56). M-preg 3tr = Mother pregnancy (3rd trimester). M-2m pp = Mother 2 months post-partum.

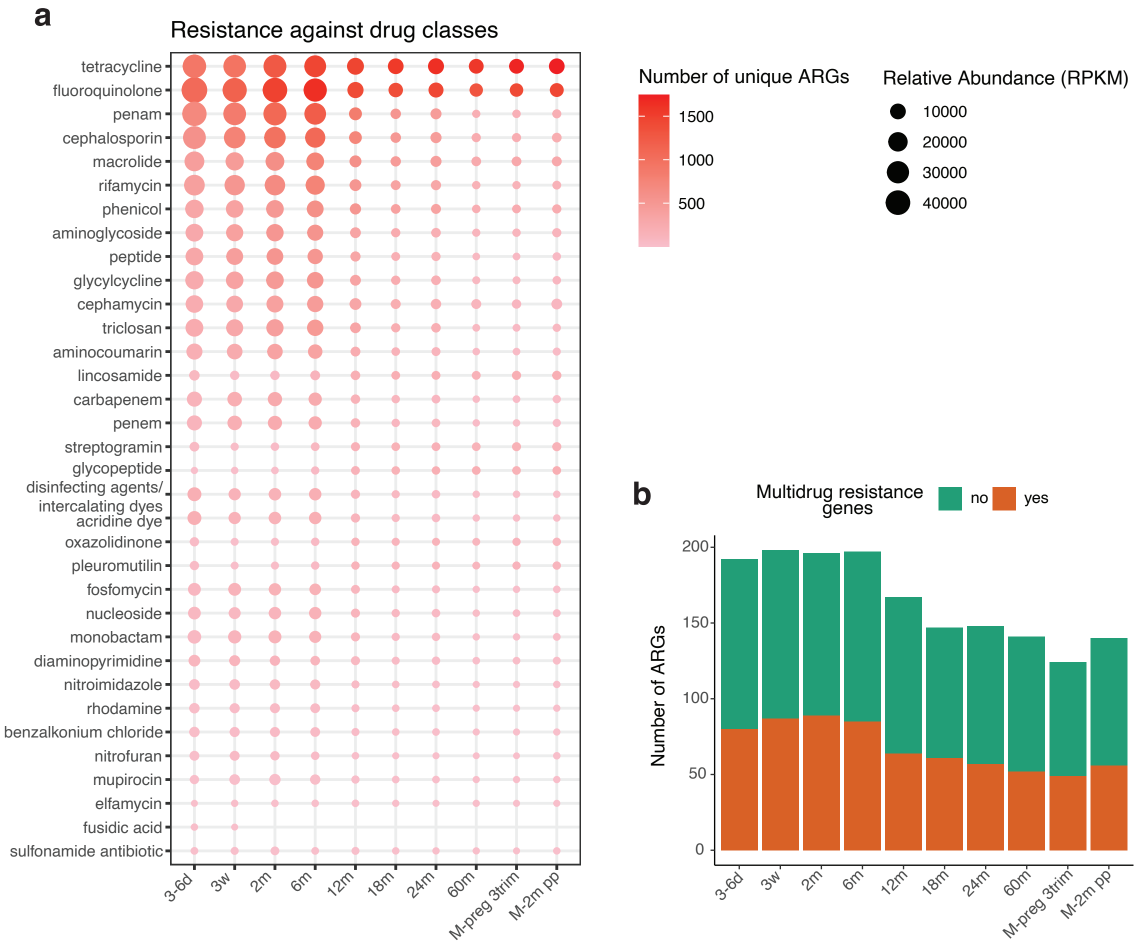

Supplementary Fig. 2│ARGs detected in faecal samples in early infancy provide resistance against a wide range of antibiotics. a, ARG genes categorized according to the antibiotic class against which they confer resistance according to CARD. The size of the dots represents the total abundance per time point and the color the number of unique ARGs. **b,** The number of ARGs per time point separated according to unique-drug or multi-drug resistance. See Supplementary Table 1 for the sample size (*N*) for each time point. M-preg 3trim = Mother pregnancy (3rd trimester). M-2m pp = Mother 2 months post-partum.

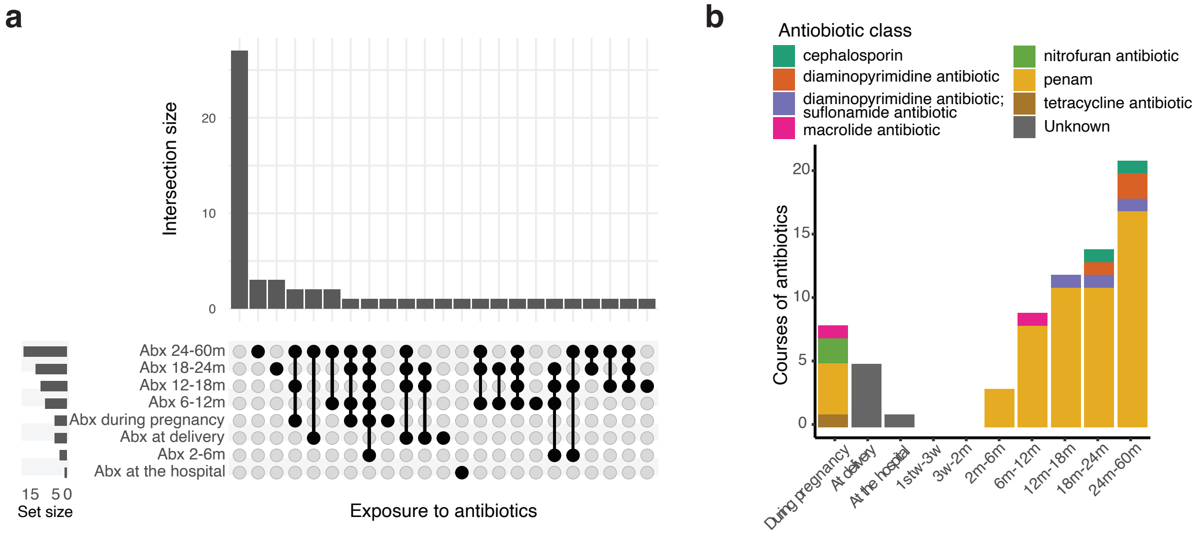

Supplementary Fig. 3│Antibiotics prescriptions in the ALADDIN cohort participants. a, Upset plot with the number of infants that were exposed to at least one antibiotic course during the specific time interval according to the Swedish Drug Registry. **b,** Antibiotic classes of the consumed antibiotics per time interval for all infants and their mothers during pregnancy.

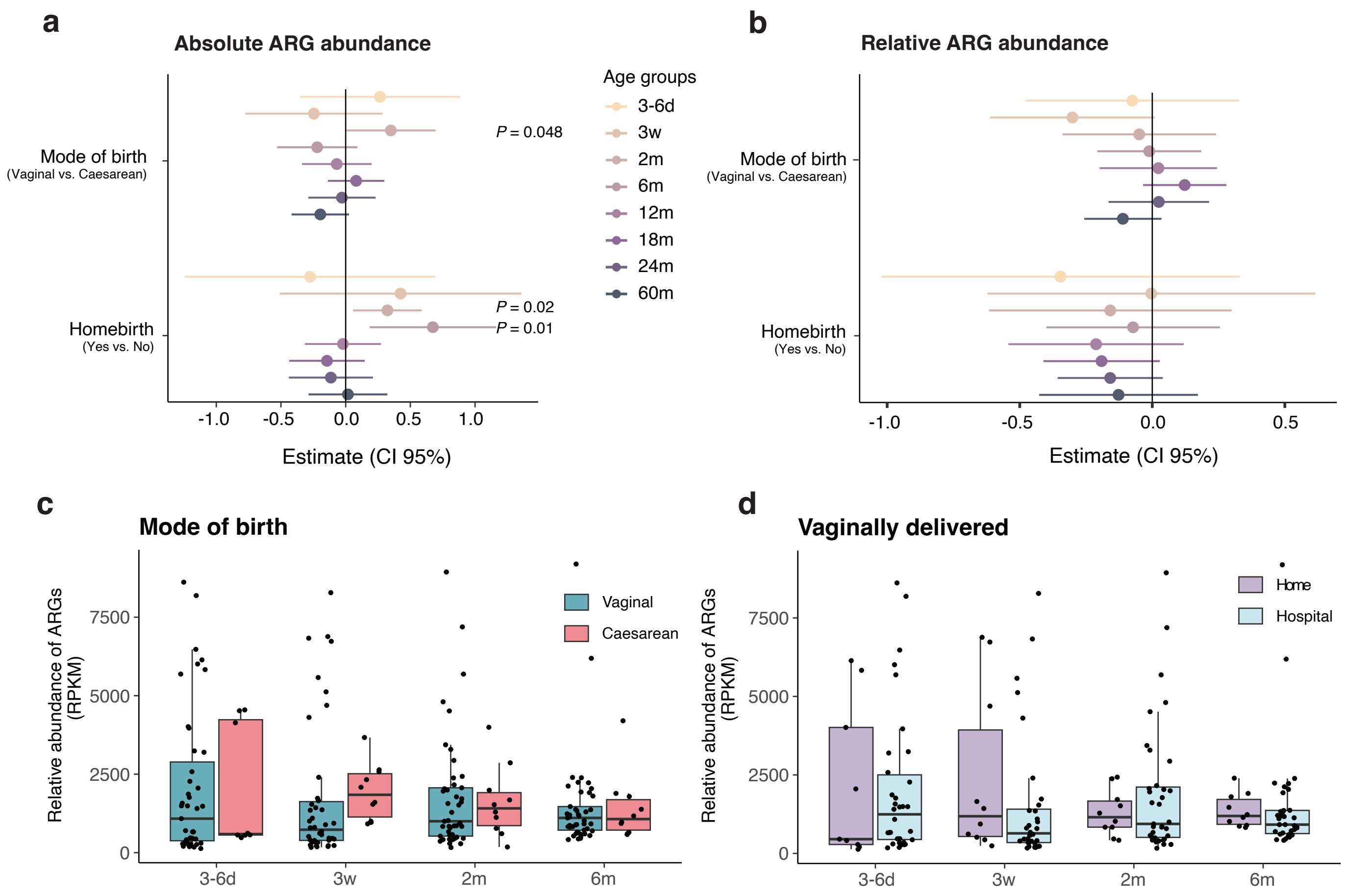

Supplementary Fig. 4│Absolute but not relative ARG abundance relates to birth mode. a,b: Effect size from linear regression models of the log transformed **a,** absolute ARG abundance and **b,** relative ARG abundance of the vaginal vs caesarean delivered infants and vaginally delivered infants born at home versus the hospital. Models were controlled for sex, family lifestyle and feeding pattern per time point. **c**, ARG relative abundance in Reads Per Kilobase per Million mapped reads (RPKM) in early life split by mode of birth and **d**, by home or hospital delivery of vaginally delivered infants. Statistics are based on linear regression, controlled for sex, family lifestyle and feeding pattern. **a,b**: Horizontal lines indicate the median; box boundaries indicate the interquartile range; whiskers represent values within 1.5× the interquartile range of the first and third quartiles. Dots represent the individual data points.

a

b

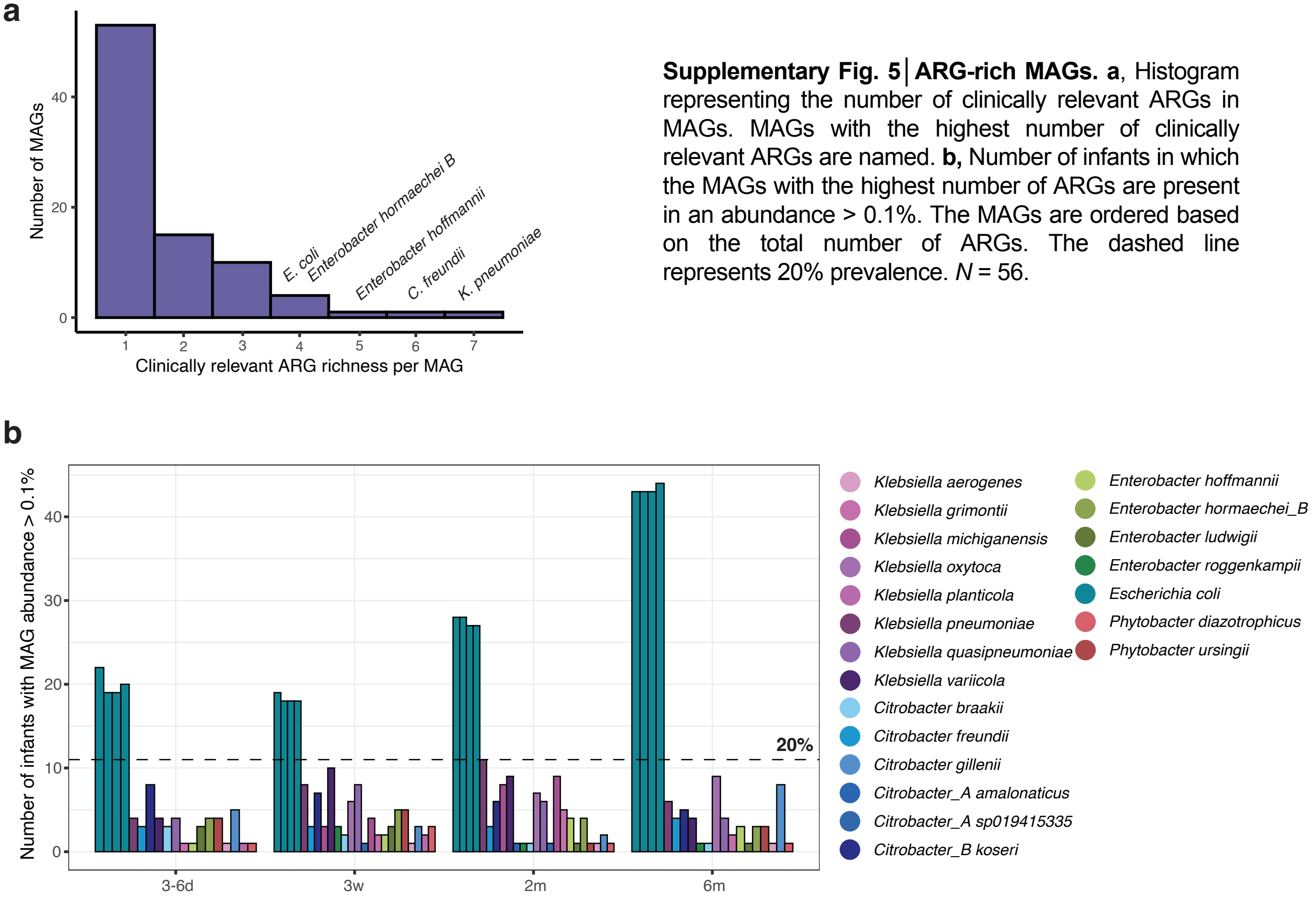

Supplementary Fig. 5│ARG-rich MAGs. a, Histogram representing the number of clinically relevant ARGs in MAGs. MAGs with the highest number of clinically relevant ARGs are named. **b,** Number of infants in which the MAGs with the highest number of ARGs are present in an abundance > 0.1%. The MAGs are ordered based on the total number of ARGs. The dashed line represents 20% prevalence. *N* = 56.

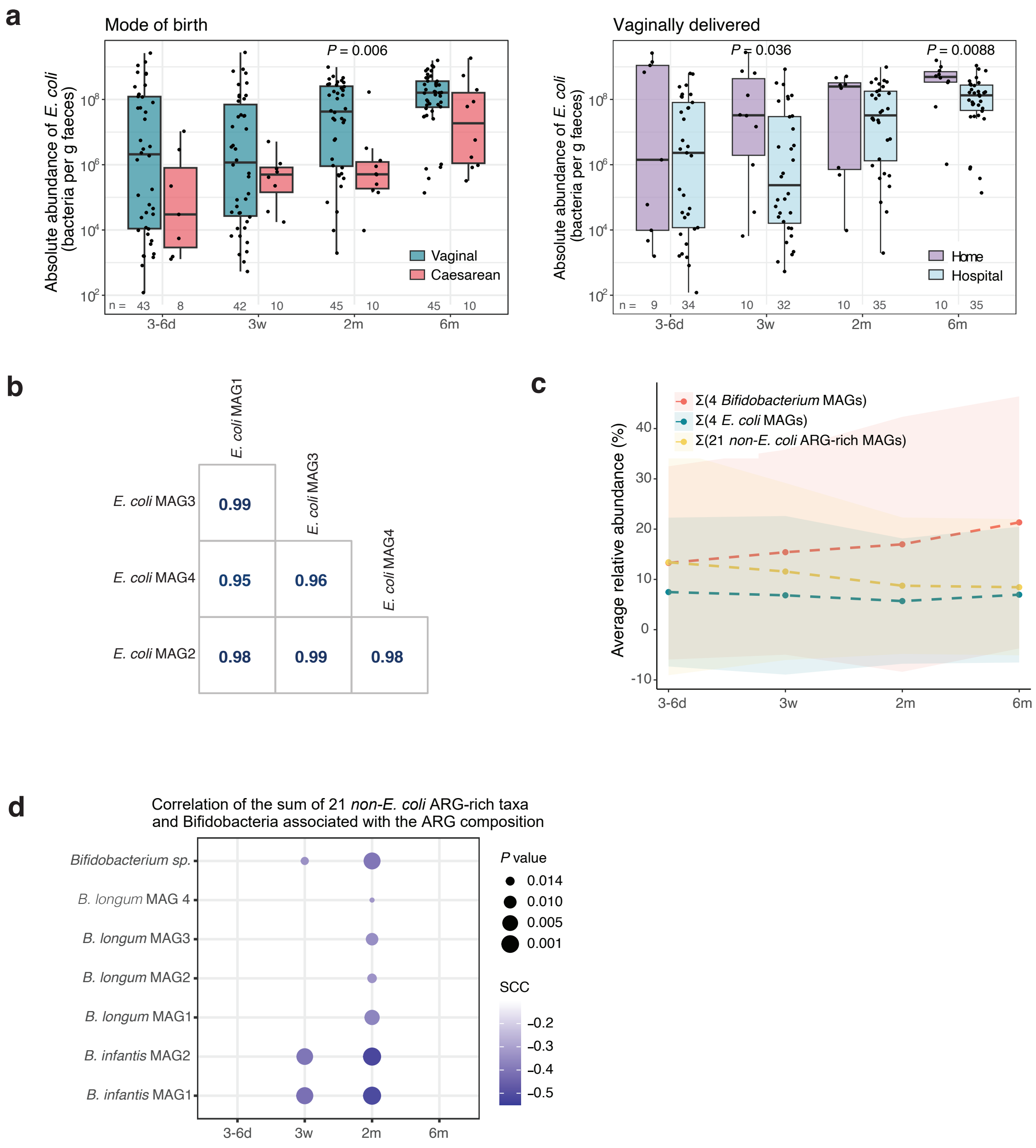

Supplementary Fig. 6 | Factors influencing *E. coli* and other ARG-rich taxa abundance in early life. a, Absolute abundance of *E. coli* during the first 6 months of life based on birth mode and birth location for the vaginally born infants. Horizontal lines indicate the median; box boundaries indicate the interquartile range; whiskers represent values within 1.5x the interquartile range of the first and third quartiles. Dots represent the individual data points. Statistics are based on two-sided Wilcoxon rank-sum test. **b**, Numbers inside the quadrants indicate the Spearman correlation coefficients between the four *E. coli* MAGs across all infant samples. **c**, Average relative abundance across time of the sum of the four *E. coli* MAGs, the sum of the four *Bifidobacterium* MAGs (*B. infantis* MAG1, *B. infantis* MAG2, *B. longum* MAG2, *B. longum* MAG3) and the sum of the 21 non-*E. coli* ARG-rich taxa for all infants that were fully breastfed at 2 months (n = 46). Line represents the average and the ribbon represents the standard deviation. **d**, Spearman correlation of relative abundance of the sum of the 21 non-*E. coli* ARG-rich taxa vs. the *Bifidobacterium* MAGs associated with ARG composition. SCC: Spearman Correlation Coefficient.

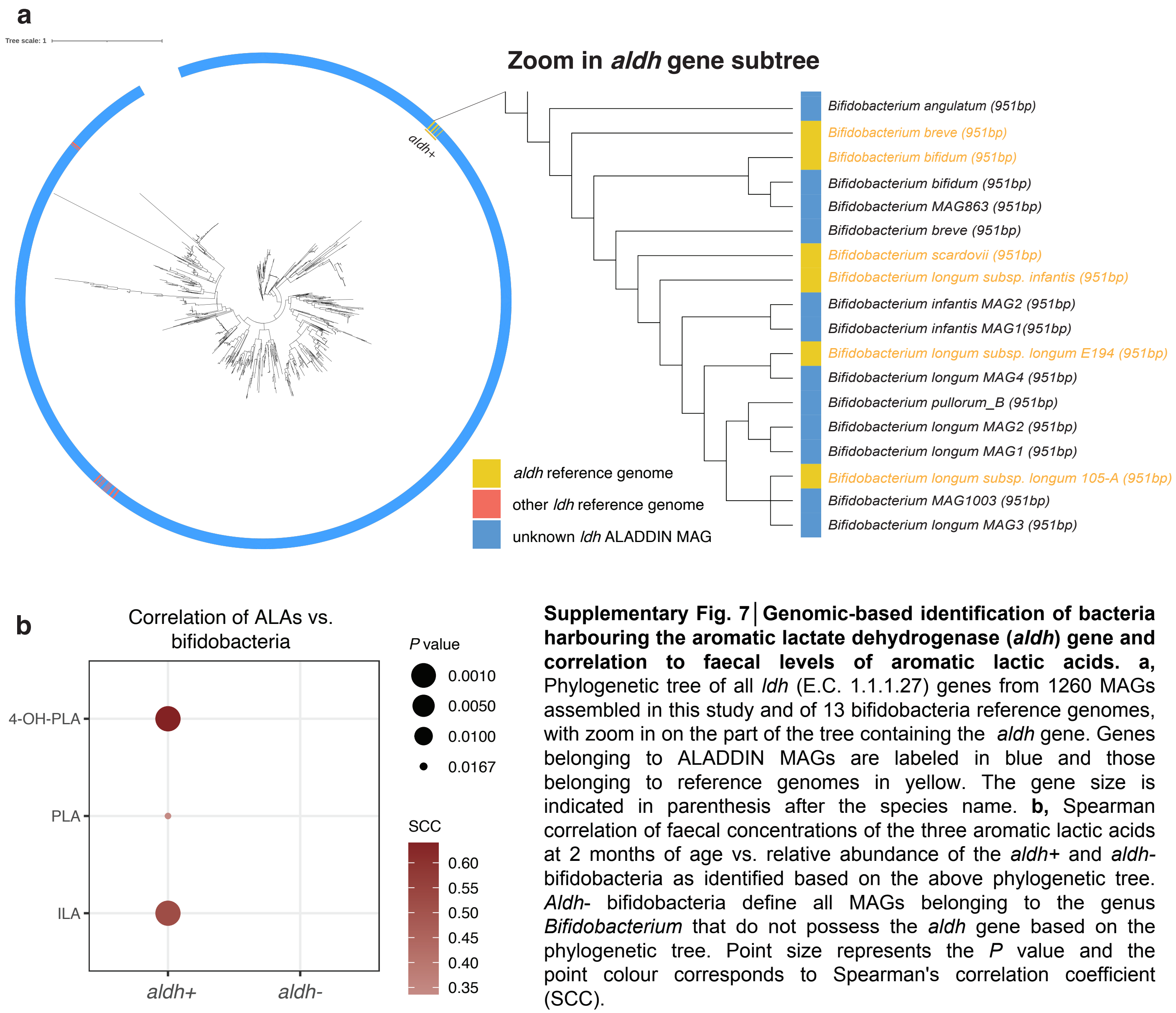

Supplementary Fig. 7│Genomic-based identification of bacteria harbouring the aromatic lactate dehydrogenase (*aldh*) gene and correlation to faecal levels of aromatic lactic acids. a, Phylogenetic tree of all *ldh* (E.C. 1.1.1.27) genes from 1260 MAGs assembled in this study and of 13 bifidobacteria reference genomes, with zoom in on the part of the tree containing the *aldh* gene. Genes belonging to ALADDIN MAGs are labeled in blue and those belonging to reference genomes in yellow. The gene size is indicated in parenthesis after the species name. **b,** Spearman correlation of faecal concentrations of the three aromatic lactic acids at 2 months of age vs. relative abundance of the *aldh*+ and *aldh*- bifidobacteria as identified based on the above phylogenetic tree. *Aldh*- bifidobacteria define all MAGs belonging to the genus *Bifidobacterium* that do not possess the *aldh* gene based on the phylogenetic tree. Point size represents the *P* value and the point colour corresponds to Spearman's correlation coefficient (SCC).

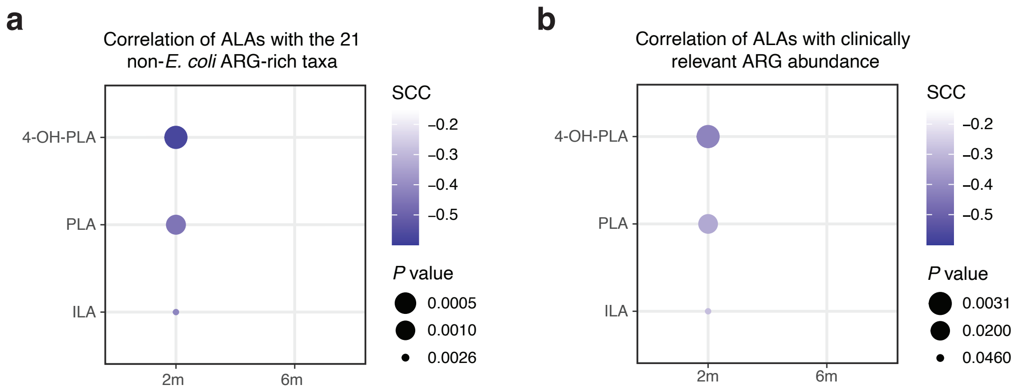

Supplementary Fig. 8 │The aromatic lactic acids levels inversely correlate to other ARG-rich taxa besides *E. coli* and to clinically relevant ARGs. a, Spearman correlation of faecal concentrations of the three aromatic lactic acids at 2 and 6 months of age vs. the sum of the relative abundance of 21 non-*E. coli* ARG-rich taxa. **b,** Spearman correlation of faecal concentrations of the three aromatic lactic acids at 2 and 6 months of age vs. the relative abundance of the clinically relevant ARGs at the same time points. Point size represents the *P* value and the point colour corresponds to Spearman's correlation coefficient (SCC).

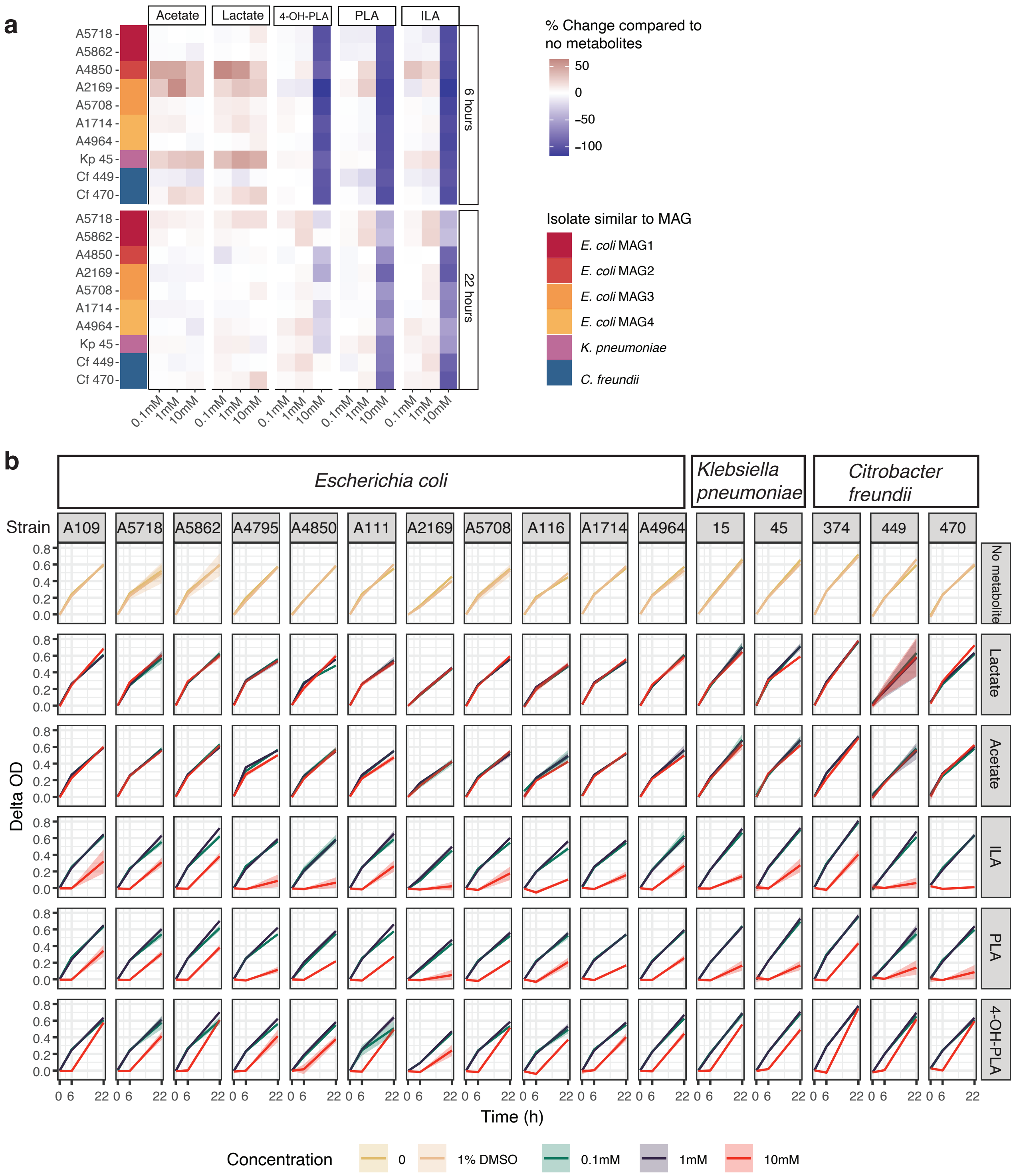

Supplementary Fig. 9│Effect of metabolites on growth of in vitro cultivated infant isolates. a, Growth difference in the presence of three concentrations of the metabolites acetate, lactate, 4-OH-PLA, PLA, ILA compared to no metabolites of seven additional *E. coli*, one *K. pneumoniae* and two *C. freundii* that are not displayed in Fig 4g. The colour of the heatmap represents the average percentage change of three independent biological replicates, each tested in triplicates. **b,** Growth curves of the eleven *E. coli,* two *K. pneumoniae* and three *C. freundii* strains grown under different concentrations of lactate, acetate, ILA, PLA and 4-OH-PLA. Delta OD represents the OD600 for individual cultures after subtracting the OD600 for no growth controls (same isolate but treated with antibiotics). The line represents the average of the three independent biological replicates and the ribbon depicts the standard deviation.

Supplementary Table 1 | Demographic characteristics of the ALADDIN study population.

**Infants**	***N***
Sex	Female = 32
	Male = 24
Lifestyle	Anthroposophic = 21
	Non-anthroposophic = 35
Birth mode	Vaginal delivery = 46
	Caesarean section = 10
Homebirth (among vaginally delivered)	Yes = 10
	No = 36
Older siblings	Yes = 35
	No = 20
Pets at 2 months	Yes = 24
	No = 32
Farm animals at 2 months	Yes = 18
	No = 38
Breastfeeding	
At 2 months	Fully = 46
	Partially = 8
	No = 2
At 6 months	Fully = 11
	Partially = 34
	Not = 10
At 12 months	Fully = 1
	Partially = 16
Introduction of solid foods at 6 months	Yes = 37
	No = 9
Going to daycare	
< 2 months	Yes = 4
	No = 52
< 6 months	Yes = 10
	No = 46
<12 months	Yes = 29
	No = 27
< 18 months	Yes = 48
	No = 8
< 24 months	Yes = 52
	No = 4
< 60 months	Yes = 52
	No = 4
Antibiotics	
between 0 and <6 months of age	Yes = 4
	No = 52
between 6 and <12 months of age	Yes = 9
	No = 47
between 12 and <18 months of age	Yes = 11
	No = 45
between 18 and <24 months of age	Yes = 13
	No = 43
between 24 and <60 months of age	Yes =18
	No = 38
**Mothers**	
Antibiotics during pregnancy	Yes = 5
	No = 51
Antibiotics at delivery	Yes = 5
	No = 50
Organic diet during pregnancy	Yes = 20
	No = 36
Organic diet during breastfeeding	Yes = 21
	No = 35

Supplementary Table 2 | Temporal differences in relative abundance of ARGs amongst ALADDIN children and their mothers. Z scores were calculated with a two-sided Dunn's test and *P* values were adjusted based on Benjamini-Hochberg correction for multiple testing. *N* = 56 mother-infant dyads. M-preg 3tr = Mother pregnancy (3rd trimester). M-2m pp = Mother 2 months post-partum.

Comparison	Z	*P adj*
3-6d - 3w	0.062	0.475
3-6d - 2m	-0.765	0.256
3-6d - 6m	-1.148	0.153
3-6d - 12m	-4.213	**2.36E-05**
3-6d - 18m	-6.752	**1.93E-11**
3-6d - 24m	6.606	**4.68E-11**
3-6d - 60m	8.301	**2.90E-16**
3-6d - M-preg 3tr	8.693	**1.59E-17**
3-6d - M-2m pp	7.167	**1.15E-12**
3w - 2m	0.832	0.240
3w - 6m	-1.217	0.144
3w - 12m	-4.171	**2.62E-05**
3w - 18m	-6.723	**2.22E-11**
3w - 24m	-6.576	**5.44E-11**
3w - 60m	8.280	**3.09E-16**
3w - M-preg 3tr	8.674	**1.57E-17**
3w - M-2m pp	7.140	**1.31E-12**
2m - 6m	-0.391	0.373
2m - 12m	-5.079	**3.88E-07**
2m - 18m	-7.663	**3.14E-14**
2m - 24m	-7.519	**8.90E-14**
2m - 60m	9.242	**1.36E-19**
2m - M-preg 3tr	9.647	**5.70E-21**
2m - M-2m pp	8.091	**1.33E-15**
6m - 12m	-5.472	**4.78E-08**
6m - 18m	-8.054	**1.64E-15**
6m - 24m	-7.911	**4.78E-15**
6m - 60m	-9.633	**4.35E-21**
6m - M-preg 3tr	10.039	**2.30E-22**
6m - M-2m pp	8.484	**7.01E-17**
12m - 18m	2.619	**7.09E-03**
12m - 24m	2.451	**0.011**
12m - 60m	4.205	**2.35E-05**
12m - M-preg 3tr	4.589	**4.37E-06**
12m - M-2m pp	3.026	**2.07E-03**
18m - 24m	-0.179	0.439
18m - 60m	1.579	0.078
18m - M-preg 3tr	1.949	**0.037**
18m - M-2m pp	0.393	0.381
24m - 60m	1.765	0.055
24m - M-preg 3tr	2.138	**0.024**
24m - M-2m pp	0.575	0.318
60m - M-preg 3tr	0.363	0.375
60m - M-2m pp	-1.193	0.146
M-preg 3trim - M-2m pp	1.563	0.078

Supplementary Table 3 | Temporal differences in absolute abundance of ARGs amongst ALADDIN children and their mothers. Z scores were calculated with a two-sided Dunn's test and *P* values were adjusted based on Benjamini-Hochberg correction for multiple testing. *N* = 56 mother-infant dyads. M-2m pp = Mother 2 months post-partum.

Comparison	Z	*P adj*
3-6d - 3w	0.083	0.480
3-6d - 2m	-3.129	**2.43E-03**
3-6d - 6m	-5.724	**3.11E-08**
3-6d - 12m	-0.150	0.466
3-6d - 18m	-0.965	0.241
3-6d - 24m	0.427	0.402
3-6d - 60m	1.217	0.212
3-6d - M-2m pp	-1.018	0.232
3w - 2m	3.228	**1.87E-03**
3w - 6m	-5.839	**1.89E-08**
3w - 12m	-0.065	0.474
3w - 18m	-0.885	0.260
3w - 24m	-0.343	0.425
3w - 60m	1.138	0.219
3w - M-2m pp	-1.108	0.219
2m - 6m	-2.550	**0.014**
2m - 12m	-3.383	**1.17E-03**
2m - 18m	-4.190	**5.59E-05**
2m - 24m	-3.662	**4.51E-04**
2m - 60m	4.443	**2.00E-05**
2m - M-2m pp	2.196	**0.032**
6m - 12m	-6.075	**5.57E-09**
6m - 18m	-6.885	**5.18E-11**
6m - 24m	-6.359	**1.22E-09**
6m - 60m	-7.143	**1.64E-11**
6m - M-2m pp	4.852	**3.14E-06**
12m - 18m	0.845	0.265
12m - 24m	0.287	0.436
12m - 60m	1.105	0.211
12m - M-2m pp	-1.207	0.205
18m - 24m	-0.559	0.357
18m - 60m	0.259	0.434
18m - M-2m pp	-2.043	**0.043**
24m - 60m	0.820	0.265
24m - M-2m pp	-1.493	0.136
60m - M-2m pp	-2.302	**0.026**

Supplementary Table 4 | Comparison of ARG abundance between abx-exposed and abx-naïve infants from first week of life to 5 years of age in ALADDIN enrolled children. Estimates and *P* values are based on two-sided Wilcoxon rank-test. Infants with at least one exposure to abx before the described period were considered abx-exposed, otherwise they were considered abx-naïve. *N* is provided in Supplementary Table 1.

Data type	Comparison	Time point	Estimate	*P* value
ARG relative abundance	abx before 1st sample vs naïve	3-6d	51.882	0.712
	abx before 1st sample vs naïve	3w	-166.119	0.559
	abx before 1st sample vs naïve	2m	-379.639	0.255
	abx before 1st sample vs naïve	6m	312.875	0.064
	abx before 1st sample vs naïve	12m	68.768	0.369
	abx before 1st sample vs naïve	18m	51.360	0.123
	abx before 1st sample vs naïve	24m	1.739	0.984
	abx before 1st sample vs naïve	60m	-44.125	0.123
	at least 1 abx before 12 months vs naïve	12m	-107.986	0.227
	at least 1 abx before 18 months vs naïve	18m	-31.281	0.281
	at least 1 abx before 24 months vs naïve	24m	-34.243	0.212
ARG absolute abundance	abx before 1st sample vs naïve	3-6d	110354	0.900
	abx before 1st sample vs naïve	3w	104110	0.728
	abx before 1st sample vs naïve	2m	-362719	0.678
	abx before 1st sample vs naïve	6m	470025	0.767
	abx before 1st sample vs naïve	12m	2640323	0.415
	abx before 1st sample vs naïve	18m	534017	0.123
	abx before 1st sample vs naïve	24m	375180	0.183
	abx before 1st sample vs naïve	60m	-106477	0.686
	at least 1 abx before 12 months vs naïve	12m	-721530	0.091
	at least 1 abx before 18 months vs naïve	18m	34474	0.921
	at least 1 abx before 24 months vs naïve	24m	133521	0.721

Supplementary Table 5 | Temporal differences in ARG Shannon Diversity amongst ALADDIN children and their mothers. Z scores were calculated with a two-sided Dunn's test and *P* values were adjusted based on Benjamini-Hochberg correction for multiple testing. *N* = 56 mother-infant dyads. M-preg 3tr = Mother pregnancy (3rd trimester). M-2m pp = Mother 2 months post-partum.

Comparison	Z	*P adj*
3-6d - 3w	1.262	0.133
3-6d - 2m	-1.171	0.147
3-6d - 6m	-3.378	**7.47E-04**
3-6d - 12m	-0.946	0.204
3-6d - 18m	-2.806	**4.52E-03**
3-6d - 24m	4.013	**7.93E-05**
3-6d - 60m	4.707	**5.14E-06**
3-6d - M-preg 3tr	4.872	**2.49E-06**
3-6d - M-2m pp	4.334	**2.35E-05**
3w - 2m	2.462	**0.012**
3w - 6m	-4.680	**5.37E-06**
3w - 12m	0.340	0.384
3w - 18m	-1.534	0.083
3w - 24m	-2.743	**5.27E-03**
3w - 60m	3.445	**6.11E-04**
3w - M-preg 3tr	3.606	**3.69E-04**
3w - M-2m pp	3.065	**2.04E-03**
2m - 6m	-2.250	**0.020**
2m - 12m	-2.164	**0.024**
2m - 18m	-4.054	**7.55E-05**
2m - 24m	-5.291	**3.04E-07**
2m - 60m	5.992	**6.65E-09**
2m - M-preg 3tr	6.167	**2.62E-09**
2m - M-2m pp	5.618	**5.42E-08**
6m - 12m	-4.423	**1.68E-05**
6m - 18m	-6.304	**1.31E-09**
6m - 24m	-7.551	**2.43E-13**
6m - 60m	-8.242	**1.91E-15**
6m - M-preg 3tr	8.426	**8.03E-16**
6m - M-2m pp	7.878	**2.49E-14**
12m - 18m	1.909	**0.040**
12m - 24m	3.142	**1.64E-03**
12m - 60m	3.856	**1.44E-04**
12m - M-preg 3tr	4.021	**8.14E-05**
12m - M-2m pp	3.471	**5.84E-04**
18m - 24m	1.219	0.139
18m - 60m	1.938	**0.038**
18m - M-preg 3tr	2.094	**0.027**
18m - M-2m pp	1.546	0.083
24m - 60m	0.728	0.262
24m - M-preg 3tr	0.879	0.219
24m - M-2m pp	0.329	0.380
60m - M-preg 3tr	0.147	0.441
60m - M-2m pp	-0.401	0.369
M-preg 3tr - M-2m pp	0.551	0.319

Supplementary Table 6 | Comparison of ARG diversity between abx-exposed and abx-naïve infants from first week of life to 5 years of age in ALADDIN enrolled children. Estimates and *P* value are based on two-sided Wilcoxon rank-test. Infants with at least one exposure to abx before the described period were considered abx-exposed, otherwise they were considered abx-naïve. *N* is provided in Supplementary Table 1.

Data type	Comparison	Time point	Estimate	*P* value
ARG shannon diversity	abx before 1st sample vs naïve	3-6d	0.166	0.602
	abx before 1st sample vs naïve	3w	0.091	0.758
	abx before 1st sample vs naïve	2m	0.021	0.959
	abx before 1st sample vs naïve	6m	0.676	**1.17E-03**
	abx before 1st sample vs naïve	12m	0.179	0.654
	abx before 1st sample vs naïve	18m	0.493	0.083
	abx before 1st sample vs naïve	24m	0.100	0.611
	abx before 1st sample vs naïve	60m	6.24E-03	0.992
	at least 1 abx before 12 months vs naïve	12m	0.033	0.935
	at least 1 abx before 18 months vs naïve	18m	0.404	0.149
	at least 1 abx before 24 months vs naïve	24m	0.118	0.532
ARG richness	abx before 1st sample vs naïve	3-6d	-1.000	0.907
	abx before 1st sample vs naïve	3w	5.000	0.659
	abx before 1st sample vs naïve	2m	-3.252	0.591
	abx before 1st sample vs naïve	6m	5.000	0.347
	abx before 1st sample vs naïve	12m	-3.000	0.687
	abx before 1st sample vs naïve	18m	9.764	0.171
	abx before 1st sample vs naïve	24m	-2.000	0.702
	abx before 1st sample vs naïve	60m	2.03E-05	0.941
	at least 1 abx before 12 months vs naïve	12m	-3.000	0.613
	at least 1 abx before 18 months vs naïve	18m	2.000	0.547
	at least 1 abx before 24 months vs naïve	24m	3.11E-06	0.953

Supplementary Table 7 | Effect of environmental variables on ARG beta-diversity dissimilarity from first week of life to 5 years of age in ALADDIN enrolled children. Tested using PERMANOVA and accounting for sex, birth mode, and family lifestyle. *P* values were corrected per time point using Benjamini-Hochberg correction for multiple testing. df = degrees of freedom, SS = Sum of squares, *N* = number of infants.

Variable	df	SS	R2	F	*P* value	*N*	*P adj*	Time point
Child sex	1	0.334	0.022	1.167	0.248	51	0.612	3-6d
	1	0.412	0.029	1.640	0.159	52	0.266	3w
	1	0.242	0.024	1.348	0.215	55	0.502	2m
	1	0.294	0.046	2.678	0.026	55	0.121	6m
	1	0.301	0.026	1.398	0.210	56	0.490	12m
	1	0.084	7.85E-03	0.418	0.905	55	0.959	18m
	1	0.200	0.019	1.031	0.361	56	0.670	24m
	1	0.148	0.016	0.868	0.631	55	0.827	60m
Birth mode	1	1.133	0.075	3.960	4.00E-03	51	**0.044**	**3-6d**
	1	1.307	0.093	5.206	4.00E-03	52	**0.044**	**3w**
	1	0.659	0.064	3.677	4.00E-03	55	0.056	2m
	1	0.421	0.066	3.829	4.00E-03	55	0.056	6m
	1	0.113	0.010	0.525	0.762	56	0.860	12m
	1	0.171	0.016	0.851	0.488	55	0.805	18m
	1	0.174	0.017	0.895	0.486	56	0.702	24m
	1	0.217	0.023	1.268	0.196	55	0.809	60m
Homebirth	1	0.226	0.019	0.795	0.495	43	0.758	3-6d
	1	0.116	0.010	0.444	0.821	42	0.821	3w
	1	0.102	0.013	0.576	0.709	45	0.764	2m
	1	0.072	0.016	0.682	0.647	45	0.874	6m
	1	0.105	0.011	0.528	0.806	46	0.860	12m
	1	0.131	0.015	0.667	0.653	45	0.805	18m
	1	0.219	0.025	1.106	0.311	46	0.670	24m
	1	0.146	0.019	0.840	0.637	46	0.827	60m
Antibiotics to mother at delivery	1	0.095	6.32E-03	0.327	0.943	50	0.943	3-6d
	1	0.118	8.50E-03	0.487	0.778	51	0.821	3w
	1	0.120	0.012	0.654	0.659	54	0.764	2m
	1	0.114	0.018	1.019	0.337	54	0.674	6m
	1	0.336	0.029	1.695	0.125	55	0.490	12m
	1	0.164	0.016	0.835	0.504	54	0.805	18m
	1	0.311	0.030	1.606	0.129	55	0.335	24m
	1	0.125	0.014	0.729	0.763	54	0.827	60m
Abx exposure before 12m	1	0.129	0.011	0.642	0.683	56	0.860	12m
Abx exposure before 18m	1	0.226	0.021	1.169	0.273	55	0.762	18m
Abx exposure before 24m	1	0.125	0.012	0.632	0.747	56	0.883	24m
Abx exposure before 60m	1	0.146	0.016	0.843	0.607	55	0.827	60m
Organic diet during pregnancy	1	0.217	0.014	0.757	0.551	51	0.758	3-6d
	1	0.233	0.017	0.964	0.407	52	0.560	3w
	1	0.174	0.017	0.971	0.419	55	0.627	2m
	1	0.047	7.33E-03	0.421	0.832	55	0.874	6m
	1	0.228	0.019	1.141	0.302	56	0.529	12m
	1	0.306	0.029	1.593	0.137	55	0.594	18m
	1	0.157	0.015	0.798	0.564	56	0.733	24m
	1	0.152	0.016	0.878	0.591	55	0.827	60m
Mother follows organic diet during breastfeeding	1	0.202	0.013	0.703	0.637	51	0.779	3-6d
	1	0.368	0.026	1.542	0.156	52	0.266	3w
	1	0.253	0.025	1.421	0.189	55	0.502	2m
	1	0.057	8.88E-03	0.511	0.764	55	0.874	6m
	1	0.360	0.030	1.829	0.109	56	0.490	12m
	1	0.130	0.012	0.664	0.662	55	0.805	18m

	1	0.350	0.033	1.818	0.070	56	0.335	24m
	1	0.126	0.014	0.729	0.753	55	0.827	60m
Exclusive breastfeeding at 2m	1	0.506	0.049	2.928	0.017	55	0.119	2m
Breastfeeding at 2m	2	0.563	0.055	1.606	0.105	55	0.368	2m
Breastfeeding at 6m	2	0.266	0.042	1.219	0.257	54	0.600	6m
Breastfeeding at 12m	2	0.330	0.028	0.817	0.625	56	0.860	12m
Solid food at 6m	1	0.071	0.011	0.637	0.693	55	0.874	6m
Family lifestyle	1	0.335	0.022	1.176	0.278	51	0.612	3-6d
	1	0.722	0.051	2.990	0.031	52	0.114	3w
	1	0.169	0.016	0.940	0.418	55	0.627	2m
	1	0.051	7.99E-03	0.462	0.874	55	0.874	6m
	1	0.100	8.60E-03	0.459	0.860	56	0.860	12m
	1	0.072	6.73E-03	0.354	0.959	55	0.959	18m
	1	0.074	7.10E-03	0.378	0.952	56	0.952	24m
	1	0.096	0.010	0.559	0.921	55	0.921	60m
Exposure to pets at 2m	1	0.091	8.85E-03	0.500	0.830	55	0.830	2m
	1	0.058	9.05E-03	0.521	0.822	55	0.874	6m
	1	0.136	0.012	0.677	0.595	56	0.860	12m
	1	0.217	0.020	1.117	0.293	55	0.762	18m
	1	0.180	0.017	0.916	0.437	56	0.702	24m
	1	0.283	0.031	1.661	0.055	55	0.598	60m
Exposure to farm animals at 2m	1	0.133	0.013	0.735	0.576	55	0.733	2m
	1	0.189	0.029	1.734	0.117	55	0.410	6m
	1	0.261	0.022	1.312	0.210	56	0.490	12m
	1	0.131	0.012	0.672	0.681	55	0.805	18m
	1	0.334	0.031	1.732	0.104	56	0.335	24m
	1	0.256	0.028	1.501	0.092	55	0.598	60m
Older siblings	1	0.179	0.012	0.618	0.724	50	0.796	3-6d
	1	0.384	0.028	1.598	0.150	51	0.266	3w
	1	0.361	0.036	2.022	0.080	54	0.368	2m
	1	0.060	0.010	0.555	0.792	54	0.874	6m
	1	0.618	0.053	3.168	0.024	55	0.336	12m
	1	0.134	0.013	0.685	0.655	54	0.805	18m
	1	0.409	0.039	2.127	0.029	55	0.335	24m
	1	0.127	0.014	0.731	0.752	54	0.827	60m
Going to daycare	1	0.224	0.019	1.120	0.295	56	0.529	12m
	1	0.381	0.036	2.001	0.067	55	0.594	18m
	1	0.112	0.011	0.567	0.829	56	0.898	24m
	1	0.178	0.019	1.034	0.374	55	0.827	60m

Supplementary Table 8 | Temporal differences in richness of ARG-containing MAGs amongst ALADDIN children and their mothers. Z scores were calculated with a two-sided Dunn's test and *P* values were adjusted based on Benjamini-Hochberg correction for multiple testing. *N* = 56 mother-infant dyads. M-preg 3tr = Mother pregnancy (3rd trimester). M-2m pp = Mother 2 months post-partum.

Comparison	Z	*P adj*
3-6d - 3w	-0.972	0.213
3-6d - 2m	-3.435	**1.21E-03**
3-6d - 6m	-5.175	**1.28E-06**
3-6d - 12m	2.397	**0.021**
3-6d - 18m	1.167	0.182
3-6d - 24m	-1.034	0.205
3-6d - 60m	0.354	0.428
3-6d - M-preg 3tr	0.293	0.433
3-6d - M-2m pp	-0.041	0.484
3w - 2m	2.462	**0.019**
3w - 6m	-4.211	**9.55E-05**
3w - 12m	1.414	0.148
3w - 18m	0.182	0.470
3w - 24m	0.044	0.493
3w - 60m	1.347	0.148
3w - M-preg 3tr	1.290	0.158
3w - M-2m pp	0.954	0.212
2m - 6m	-1.774	0.086
2m - 12m	-1.074	0.199
2m - 18m	-2.313	**0.025**
2m - 24m	-2.464	**0.021**
2m - 60m	3.863	**3.14E-04**
2m - M-preg 3tr	3.817	**3.38E-04**
2m - M-2m pp	3.476	**1.14E-03**
6m - 12m	-2.856	**8.05E-03**
6m - 18m	-4.086	**1.41E-04**
6m - 24m	-4.245	**9.82E-05**
6m - 60m	-5.637	**3.89E-07**
6m - M-preg 3tr	5.599	**2.43E-07**
6m - M-2m pp	5.258	**1.09E-06**
12m - 18m	1.249	0.164
12m - 24m	1.396	0.146
12m - 60m	2.807	**8.66E-03**
12m - M-preg 3tr	2.755	**9.43E-03**
12m - M-2m pp	2.413	**0.021**
18m - 24m	0.141	0.476
18m - 60m	1.551	0.130
18m - M-preg 3tr	1.494	0.138
18m - M-2m pp	1.153	0.181
24m - 60m	1.417	0.153
24m - M-preg 3tr	1.359	0.151
24m - M-2m pp	1.017	0.204
60m - M-preg 3tr	-0.064	0.497
60m - M-2m pp	-0.404	0.417
M-preg 3tr - M-2m pp	0.342	0.423

Supplementary Table 9 | Concentrations of indolelactate (ILA), phenyllactate (PLA) and 4-hydroxyphenyllactate (4-OH-PLA) measured in faecal water at 2 and 6 months of age in 56 children of the ALADDIN cohort.

	**2 months**			**6 months**		
	**Median**	**IQR**	**Max**	**Median**	**IQR**	**Max**
**Metabolites**	**in nmol/g faeces**					
ILA	60.7	72.8	325	76.5	105	375
PLA	49.8	50.7	164	61.7	61.6	250
4-OH-PLA	53.4	84.6	280	61.2	97.2	326
