# Supplementary material for: Temporal dynamics and microbial interactions shaping the gut resistome in early infancy
Source: Nat Commun. 2025 Aug 30;16:8139. doi: 10.1038/s41467-025-63401-6 (PMC12398494; doi:10.1038/s41467-025-63401-6)
Supplement: Supplementary file 2 — Description of Additional Supplementary Files [file 41467_2025_63401_MOESM2_ESM.pdf]

Description of Additional Supplementary Files

File Name: Supplementary Data 1
Description: List of all detected antibiotic resistance genes identified in the cohort faecal samples according to CARD.

File Name: Supplementary Data 2
Description: Annotation of antibiotic resistance genes (ARGs) to the cohort specific metagenome-assembled genomes (MAGs).

File Name: Supplementary Data 3
Description: List of virulence genes per MAG based on the VFDB database.

File Name: Supplementary Data 4
Description: Assembly quality metrics for contigs assembled in each of the 547 faecal samples.
