# Supplementary material for: Temporal dynamics and microbial interactions shaping the gut resistome in early infancy
Source: Nat Commun. 2025 Aug 30;16:8139. doi: 10.1038/s41467-025-63401-6 (PMC12398494; doi:10.1038/s41467-025-63401-6)
Supplement: Supplementary file 7 — Reporting Summary [file 41467_2025_63401_MOESM7_ESM.pdf]

nature portfolio

Corresponding author(s): Susanne Brix

Last updated by author(s): Aug 12, 2025

Reporting Summary

Nature Portfolio wishes to improve the reproducibility of the work that we publish. This form provides structure for consistency and transparency in reporting. For further information on Nature Portfolio policies, see our Editorial Policies and the Editorial Policy Checklist.

Statistics

For all statistical analyses, confirm that the following items are present in the figure legend, table legend, main text, or Methods section.

n/a	Confirmed	
☐	☒	The exact sample size (*n*) for each experimental group/condition, given as a discrete number and unit of measurement
☒	☐	A statement on whether measurements were taken from distinct samples or whether the same sample was measured repeatedly
☐	☒	The statistical test(s) used AND whether they are one- or two-sided<br>*Only common tests should be described solely by name; describe more complex techniques in the Methods section.*
☐	☒	A description of all covariates tested
☐	☒	A description of any assumptions or corrections, such as tests of normality and adjustment for multiple comparisons
☐	☒	A full description of the statistical parameters including central tendency (e.g. means) or other basic estimates (e.g. regression coefficient) AND variation (e.g. standard deviation) or associated estimates of uncertainty (e.g. confidence intervals)
☐	☒	For null hypothesis testing, the test statistic (e.g. *F*, *t*, *r*) with confidence intervals, effect sizes, degrees of freedom and *P* value noted<br>*Give P values as exact values whenever suitable.*
☒	☐	For Bayesian analysis, information on the choice of priors and Markov chain Monte Carlo settings
☒	☐	For hierarchical and complex designs, identification of the appropriate level for tests and full reporting of outcomes
☒	☐	Estimates of effect sizes (e.g. Cohen's *d*, Pearson's *r*), indicating how they were calculated

Our web collection on statistics for biologists contains articles on many of the points above.

Software and code

Policy information about availability of computer code

Data collection	No software was used for data collection.
Data analysis	All analysis in the study was performed with open source tools. In more detail, metagenomic assembly was done with MEGAHIT (v1.1.1) and gene prediction with Prodigal (v2.6.3). Resistance gene identification was performed with RGI (v5.2.0) based on CARD v3.1.2. For metagenomic binning the following tools were used: Bowtie2 v2.5.0, Samtools v1.16, SemiBin v1.5.1, dRep v3..4.0, checkM2 v1.0.1, GTDB-Tk v2.4.0 (based on GTDB r220), ABRIcate v1.0.1, and coverM v0.6.1. InStrain v1.7.5 was used for strain analysis. prokka v1.14.5 was used for gene prediction and annotatio. clustal-omega v1.2.1, FastTree v2.1.11 and iTOL v7 for the ldh gene phylogenetic treet. The UPARSE pipeline was used for data processing of the amplicon sequencing data of the CIG cohort. More information on the specific parameters used to run each tool can be found in the Materials & Methods section of the manuscript.<br>Statistical analysis was performed using R v4.0.5 and packages: vegan v2.6.4, dunn.test v1.3.5, stats v4.2.1.

For manuscripts utilizing custom algorithms or software that are central to the research but not yet described in published literature, software must be made available to editors and reviewers. We strongly encourage code deposition in a community repository (e.g. GitHub). See the Nature Portfolio guidelines for submitting code & software for further information.

Data

Policy information about availability of data

All manuscripts must include a data availability statement. This statement should provide the following information, where applicable:

- Accession codes, unique identifiers, or web links for publicly available datasets
- A description of any restrictions on data availability
- For clinical datasets or third party data, please ensure that the statement adheres to our policy

The metagenomic raw sequencing reads and MAGs generated under ALADDIN cohort have been deposited as a BioProject with accession number PRJEB84944.16S rRNA gene amplicon sequencing data from the CIG cohort is deposited in the Sequence Read Archive (SRA) under BioProject PRJNA554596. The data on individuals' nutritional and lifestyle habits, antibiotic exposure, and faecal aromatic lactic acid concentrations are pseudonymized (coded) personal data, prohibited from public sharing.

Research involving human participants, their data, or biological material

Policy information about studies with human participants or human data. See also policy information about sex, gender (identity/presentation), and sexual orientation and race, ethnicity and racism.

Reporting on sex and gender	The sex of infants in ALADDIN cohort is reported according to biological attribute. 32 female and 24 male infants were included in the study.
Reporting on race, ethnicity, or other socially relevant groupings	No information on race or ethnicity of the subjects is reported in this study.<br>Families were classified into the lifestyle groups, anthroposophic and non-anthroposophic, based on their choice of Maternal Healthcare Center and parental responses to the questions on: choice of future type of preschool/school, view of lifestyle, and the influence of lifestyle on daily life.
Population characteristics	Faecal samples from the mothers were collected during the third trimester of pregnancy and 2 months postpartum. Faecal samples and questionnaire information from infants was received at 8 time points (3-6 days, 3 weeks, 2 months, 6 months, 12 months, 18 months, 24 months, and 60 months post of age).
Recruitment	Families were recruited at anthroposophic and conventional Maternal Healthcare Centers in the Stockholm area, Sweden. Inclusion criteria in this study were based on the existence of longitudinally collected faecal samples from 8 time points during early life of the children and twice from their mothers. Children were excluded if they were born preterm (<gestational week 36).
Ethics oversight	The ALADDIN study was approved by the Local Ethical Committee Huddinge 2002-01-07 (2002/474-01) and Regional Ethical Committee Stockholm 2010-04-30 (2010/741-32). The CIG study was approved by the Data Protection Agency (18/02459), and informed consent was obtained from all parents of infants participating in the study.

Note that full information on the approval of the study protocol must also be provided in the manuscript.

Field-specific reporting

Please select the one below that is the best fit for your research. If you are not sure, read the appropriate sections before making your selection.

☒ Life sciences ☐ Behavioural & social sciences ☐ Ecological, evolutionary & environmental sciences

For a reference copy of the document with all sections, see nature.com/documents/nr-reporting-summary-flat.pdf

Life sciences study design

All studies must disclose on these points even when the disclosure is negative.

Sample size	The sample-size was chosen based on the existence of longitudinally collected faecal samples from 8 time points during early life of the children and twice from their mothers of the ALADDIN cohort participants. A ratio between anthroposophic and non-anthroposophic families was also taken into account, resulting in 21 anthroposophic and 35 non-anthroposophic families.
Data exclusions	Shotgun sequencing samples were excluded when there was not sufficient number of high quality sequencing reads (13 samples). Faecal metabolite samples were excluded when there was no sufficient sample to perform the analysis (7 samples), or samples metabolite measurement failed QC (4 samples for ILA and 2 samples for PLA).
Replication	In vitro growth culture of the 11 E. coli strains, 2 K. pneumoniae and 3 C. freundii in the presence of the metabolites was performed in three technical replicates and three independent times.
Randomization	No randomization was performed as the study was observational.
Blinding	No blinding was performed as the study was observational.

Reporting for specific materials, systems and methods

We require information from authors about some types of materials, experimental systems and methods used in many studies. Here, indicate whether each material, system or method listed is relevant to your study. If you are not sure if a list item applies to your research, read the appropriate section before selecting a response.

Materials & experimental systems

n/a	Involved in the study
☒	☐ Antibodies
☒	☐ Eukaryotic cell lines
☒	☐ Palaeontology and archaeology
☒	☐ Animals and other organisms
☒	☐ Clinical data
☒	☐ Dual use research of concern
☒	☐ Plants

Methods

n/a	Involved in the study
☒	☐ ChIP-seq
☐	☒ Flow cytometry
☒	☐ MRI-based neuroimaging

Plants

Seed stocks	*Report on the source of all seed stocks or other plant material used. If applicable, state the seed stock centre and catalogue number. If plant specimens were collected from the field, describe the collection location, date and sampling procedures.*
Novel plant genotypes	*Describe the methods by which all novel plant genotypes were produced. This includes those generated by transgenic approaches, gene editing, chemical/radiation-based mutagenesis and hybridization. For transgenic lines, describe the transformation method, the number of independent lines analyzed and the generation upon which experiments were performed. For gene-edited lines, describe the editor used, the endogenous sequence targeted for editing, the targeting guide RNA sequence (if applicable) and how the editor was applied.*
Authentication	*Describe any authentication procedures for each seed stock used or novel genotype generated. Describe any experiments used to assess the effect of a mutation and, where applicable, how potential secondary effects (e.g. second site T-DNA insertions, mosiacism, off-target gene editing) were examined.*

Flow Cytometry

Plots

Confirm that:

☒ The axis labels state the marker and fluorochrome used (e.g. CD4-FITC).

☒ The axis scales are clearly visible. Include numbers along axes only for bottom left plot of group (a 'group' is an analysis of identical markers).

☐ All plots are contour plots with outliers or pseudocolor plots.

☒ A numerical value for number of cells or percentage (with statistics) is provided.

Methodology

Sample preparation	100-200 mg faeces was weighed accurately. The homogenized samples, diluted 1:4 in Milli-Q water, were further diluted 2.5-fold in Milli-Q water, and centrifuged at 50 x g, 4°C for 15 minutes. Supernatant was transferred to a new tube and centrifuged at 8,000 x g, 4°C for 5 minutes. The bacterial pellet was washed twice in sterile PBS + 1% BSA (>98%, Sigma-Aldrich) with centrifugation at 8,000 x g, 4°C for 5 minutes, and resuspended in sterile PBS + 1% BSA. An aliquot was diluted 150x in PBS + 1% BSA + 0.01% Tween20 + 1 mM EDTA, mixed with 1 mM DAPI and BD Liquid Counting Beads (BD Biosciences)
Instrument	FACSCanto II (BD Biosciences)
Software	Bacterial cell population gating and quantification was made using the FlowJo software.
Cell population abundance	The quantification of the bacterial cell population was made by gating the population in SSC-A/Pacific Blue and using counting beads for estimating the cell number.
Gating strategy	The bacterial cell population was defined by gating in a SSC-A/Pacific blue plot.

☒ Tick this box to confirm that a figure exemplifying the gating strategy is provided in the Supplementary Information.
